# Dietary antioxidants and CKM–depression comorbidity: a primary analysis with a secondary evaluation of all-cause mortality using six machine learning algorithms

**DOI:** 10.1186/s40001-025-03524-0

**Published:** 2025-11-27

**Authors:** Yi Tang, Zilong Yue, Juncang Wu, Hongwei Liu, Qiuwan Liu, Kangrui Zhang, Yueyu Zhang, Yu Wang, Shuaizhou Wang, Xinyi Chen, Xun He, Jie Hu, Zhinan Ye

**Affiliations:** 1 Department of Neurology, Anhui Medical University, Hefei, 230032 China; 2 Department of Neurology, The Second People’s Hospital of Hefei, Hefei, 230011 China; 3 Department of General Surgery, Guoyang Branch of Anhui Provincial Hospital, Bozhou, 233500 China; 4 Department of Neurology, Taiyuan City Central Hospital, The Ninth Clinical Medical College of Shanxi Medical University, Taiyuan, 030009 Shanxi Province China; 5 Department of Neurology, Taizhou Municipal Hospital (Taizhou University Affiliated Municipal Hospital), School of Medicine, Taizhou University, Taizhou, 318000 Zhejiang Province China

**Keywords:** Dietary antioxidants, Cardiovascular–kidney–metabolic syndrome, Depression, NHANES

## Abstract

**Background:**

We examined whether dietary antioxidant intake is associated with CKM–depression comorbidity (primary outcome) and all-cause mortality (secondary outcome) in US adults.

**Methods:**

In an observational analysis of NHANES 2007–2010 with linked mortality, we defined the primary outcome as CKM–depression comorbidity and the secondary outcome as all-cause mortality. Predictors were selected using LASSO (linear models) and Boruta (tree-based models). Six classifiers (AdaBoost, GBDT, LGBM, RF, SVM, NB) were trained. Evaluation used stratified tenfold/nested cross-validation; all preprocessing, feature selection, and SMOTE were performed inside training folds only to avoid leakage. Discrimination metrics included ROC-AUC, PR-AUC, F1, MCC, recall, specificity; calibration was assessed on out-of-fold probabilities using Brier score and calibration slope/intercept, with reliability curves. Subsequently, associations were assessed using multivariate logistic regression (for comorbidities) and Cox proportional hazards models (for all-cause mortality). We used permutation-adjusted *P*-values for multiplicity, restricted cubic splines for dose–response, and Schoenfeld residuals for the proportional-hazards assumption. *E*-values quantified residual confounding.

**Results:**

Among ML models, LGBM performed best (test AUC = 0.978) with favorable calibration. In fully adjusted models, higher anthocyanidin intake was associated with lower odds of CKM–depression comorbidity: peonidin (OR = 0.716, 95% CI 0.572, 0.897), petunidin (OR = 0.749, 95% CI 0.633, 0.885), and total anthocyanidins (OR = 0.973, 95% CI 0.959, 0.988). For all-cause mortality, Cox models showed inverse associations for petunidin (HR = 0.936, 95% CI 0.900, 0.975) and total anthocyanidins (HR = 0.993, 95% CI 0.988, 0.998), whereas peonidin was not significant after permutation adjustment (HR = 0.964, 95% CI 0.929, 1.000). Restricted cubic splines suggested monotonic inverse trends; proportional-hazards tests showed no violations.

**Conclusions:**

Specific anthocyanidins were cross-sectionally associated with lower CKM–depression comorbidity and, for petunidin and total anthocyanidins, with lower mortality hazard. Given the observational design and potential residual confounding, these findings should be interpreted as associations rather than causal or protective effects.

**Supplementary Information:**

The online version contains supplementary material available at 10.1186/s40001-025-03524-0.

## Background

Type 2 diabetes, cardiovascular disease, and chronic kidney disease interact bidirectionally and are now framed under cardio-kidney–metabolic (CKM) syndrome as a systems-level construct [1–4]. However, the psychological burden caused by multimorbidity is often underrecognized in clinical practice, especially given the characteristic long-term illness trajectories of chronic diseases [5]. Compelling epidemiologic evidence suggests a bidirectional relationship in which depressive disorders not only increase the progression of chronic diseases, such as cardiovascular disease, but also exacerbate dysfunction in the biopsychosocial domain [6, 7]. This reciprocal dynamic precipitates deleterious consequences, including diminished health-related quality of life, impaired societal participation, and escalated demands on healthcare systems [8].

Biologically, CKM and depression share inflammation and oxidative/REDOX stress pathways (together with neuroendocrine and autonomic dysregulation) [9, 10]. These shared mechanisms provide a rationale for examining dietary antioxidants, which may influence redox balance and inflammatory tone. Although antioxidant-rich diets have been linked separately to CKM components and to depressive symptoms [11–13], the joint phenotype (CKM with depression) and its relation to mortality remain understudied.

Using NHANES 2007–2010 with linked mortality, we investigated the associations between dietary antioxidants and CKM–depression comorbidity (primary outcome), and secondarily with all-cause mortality. To screen and prioritize features and enhance interpretability, we combined machine learning (LASSO, Boruta, six classifiers) with SHAP explanations, followed by multivariable logistic regression (comorbidity) and Cox models (mortality).

## Methods

The survey was organized into a three-stage methodological framework. In the initial phase, machine learning models were employed to systematically identify bioactive compounds derived from dietary antioxidants exhibiting potential association with the co-occurrence of cardiometabolic kidney disease (CKM) and depressive disorders [14, 15]. Subsequently, the second phase implemented multivariate regression analyses and mediation models to quantify the longitudinal associations between the prioritized antioxidant components and the bidirectional relationships of CKM–depression comorbidity, while controlling for demographic and lifestyle confounders—the dual analytical approach aimed to establish evidence-based linkages between nutritional biomarkers and this clinically significant comorbidity pattern. In the third stage, we used machine learning and multivariate logistic regression to identify specific bioactive components of dietary antioxidants associated with all-cause mortality.

### Study population

**Fig. 1:**
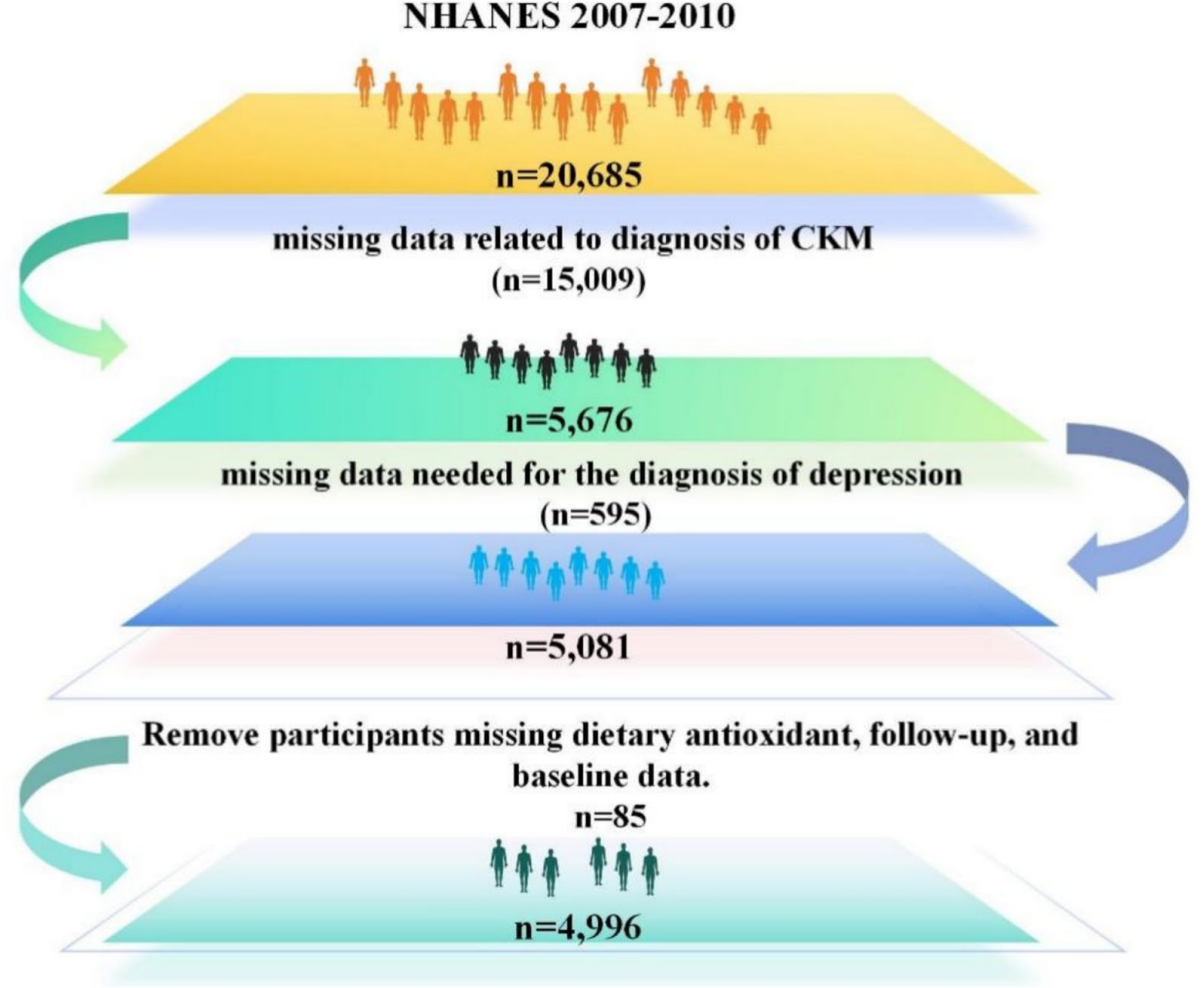
Flowchart of participants' selection. Dietary antioxidant data were missing for 20, follow-up data were missing for 17, and basic data were missing for 48

NHANES is a comprehensive research program designed to assess the dietary intake and general health of the US population, and its scope includes questionnaires and dietary assessments [16, 17]. We used information from four consecutive NHANES survey phases, from March 2007 through December 2010, to assess dietary intake and overall health. Information on the survival status of participants was collected by probabilistically matching NHANES to NDI data using a series of identifiers, including social security number and date of birth. Participants were followed up until 31 December 2019 for this study. Once a match was not found in the NDI, the participant was considered to be alive. Throughout the data collection process and subsequent analyses, the researchers could not obtain data that might have revealed the identity of the subjects. The total number of individuals eligible for the study was 20,685, of which 15,009 were missing data related to the diagnosis of CKM, and 595 were missing data needed for the diagnosis of depression. Dietary antioxidant data were missing for 20, follow-up data were missing for 17, and basic data were missing for 48 (Fig. 1).

### Assessments of CKM syndrome

CKM syndrome is characterized by the coexistence of subclinical or clinical cardiovascular disease, chronic kidney disease, and metabolic disorders [18]. Clinical cardiovascular disease includes conditions such as chronic heart failure, coronary heart disease, myocardial infarction, or stroke. Subclinical cardiovascular disease was identified by a 10-year risk of cardiovascular disease ≥ 20% or high-risk chronic kidney disease. A simplified CKM risk algorithm that includes variables such as age, sex, smoking status, blood pressure, cholesterol, diabetes mellitus, renal function, antihypertensive medication use, and statin use was used to calculate 10-year CVD risk [19]. CKD risk was stratified using estimated glomerular filtration rate thresholds and urine albumin/creatinine ratio categories based on previous literature. Metabolic disorders included overweight/obesity, abdominal obesity, prediabetes, diabetes, hypertension, dyslipidemia, and metabolic syndrome. Given the varying clinical severity of CKM syndrome manifestations, participants were categorized into four CKM stages: stage 0 (all normal); stage 1 (obesity or prediabetes only); stage 2 (at least one other metabolic disorder or CKD); stage 3 (metabolic disorder or CKD with subclinical cardiovascular disease); and stage 4 (metabolic disorder or CKD with clinical cardiovascular disease) [20]. The literature has classified CKM into three stages and has emphasized that more attention should be paid to changes in metabolic markers in stage 1 (obesity only) and stage 3 (with subclinical CVD) [21]. Therefore, in this study, CKM stages 1 through 4 were combined and categorized as having CKM.

### Assessment of depression

The PHQ-9 is a widely recognized depression assessment tool employed in clinical environments to evaluate the presence of depression. For each item on the PHQ-9, participants can select one of four responses: 0 (not at all), 1 (several days), 2 (more than half of the days), or 3 (nearly every day). The maximum total score is 27, with participants scoring above 10 considered to have major depression [22]. PHQ-9 is an instrument frequently used in healthcare settings for depression screening. It consists of questions where each is answered on a scale from 0 to 3, reflecting the frequency of depressive symptoms over the past 2 weeks. Total scores can range up to 27, and a score exceeding 10 typically indicates major depressive disorder [23].

### Dietary antioxidant intake

Information on the consumption of 44 dietary antioxidants, comprising vitamins, minerals, and polyphenols, was gathered from NHANES [24, 25]. Two 24-h dietary recall interviews were conducted at mobile examination centers, spaced 3–10 days apart. From these, the average daily intake of dietary antioxidants for each participant was determined. For specific methods of handling missing variables, please refer to the attached document.

### Covariates and candidate predictor variables

Considering the common factors associated with CKM and depression, we included general demographic characteristics (age, gender, race, family PIR, education, BMI), total energy intake, physical activity, smoking history, and drinking history as covariates [19]. A wide range of indicators were collected as potential predictors in our study, with a total of 75 candidate variables, including 69 continuous and 5 categorical variables. These variables were categorized into four main groups: demographic variables, lifestyle variables, laboratory blood indicator variables, and dietary antioxidant variables.

### Statistical analysis

To identify the main dietary antioxidants associated with CKM–depression comorbidity, an initial screening was performed using LASSO regression and Boruta algorithms, retaining the covariates screened by both algorithms [26, 27]. Subsequently, the covariates were investigated using a variance inflation factor (VIF) approach, and variables with a VIF of more than ten were excluded from subsequent machine learning (ML) modeling. To alleviate the class imbalance between the comorbidity and non-comorbidity groups, Smote sampling techniques were applied. For specific details and comparisons with the original model, please refer to Supplementary Tables 1 and 2. Six different ML models were subsequently developed: adaptive boosting (AdaBoost), gradient boosting decision tree (GBDT), light gradient boosting machine (LGBM), naive Bayes (NB), random forest (RF), and support vector machine (SVM). Area under the curve (AUC) is the main metric for evaluating models. Other performance metrics included accuracy, prevalence, sensitivity, F1-score, Matthews association coefficient (MCC), precision (positive predictive value), false negative rate (FNR), and false positive rate (FPR). A tenfold cross-validation was then used to assess the stability of the best model. A Shapley Additive exPlanation (SHAP) analysis was performed on the most validated models to assess the significance of features and to identify dietary antioxidants significantly associated with CKM and depressive comorbidity.

Weighted multivariate logistic regression analyses quantified the strength of these associations, along with smoothed curve fitting, threshold effects analyses, mediation effects analyses, and subgroup analyses to ensure stability of the relationships. Afterward, we performed a weighted Cox regression analysis of the three dietary antioxidants screened by machine learning with all-cause mortality and plotted Kaplan–Meier survival curves. Additionally, we validated the results of multiple comparisons using empirical *P*-values calculated via randomization exchange for both logistic regression and Cox regression, employing *E*-value analysis to quantify unadjusted confounding factors.

All statistical analyses were performed using R 4.3.2 (http://www.r-project.org), with *P*-values less than 0.05 indicating statistical significance. Continuous variables were expressed as medians and trinomials and were compared using the Kruskal–Wallis test. Categorical variables were expressed as frequencies and percentages and were compared using the Chi-square test.

## Results

### Baseline characteristics

As shown in Table 1, this study included 4,996 eligible participants, of whom 4,634 were assigned to the no comorbidity group and 362 to the CKM depression comorbidity group. Baseline characteristics showed that subjects had a mean age of 50.278 years, were predominantly female (51.02%), and non-Hispanic Whites were the largest racial group (47.88%). We identified statistically significant variations (*P* < 0.05) in sex, racial background, education level, smoking, family PIR, leukocyte levels, and neutrophil counts. The non-comorbidity group exhibited a higher mean family poverty–income ratio (PIR) of 3.042 compared to 2.005 in the comorbidity group, suggesting superior socioeconomic status among non-comorbid individuals. In terms of clinical presentation, the mean PHQ-9 score for the cohort was 3.266, with 386 subjects meeting diagnostic criteria for depression.

**Table 1:** Weighted basic characteristics after initial screening of participants with both CKM and depression

CKM–depression comorbidity			
Characteristics	No	Yes	*P* value
Age (years)	47.352 (46.514, 48.189)	47.280 (45.987, 48.572)	0.9187
Family PIR	3.042 (2.953, 3.130)	2.005 (1.720, 2.289)	< 0.0001
BMI (kg/m^2^)	26.997 (26.706, 27.287)	26.756 (25.800, 27.711)	0.6647
Total energy intake (kcal)	2163.980 (2124.020, 2203.939)	2026.780 (1920.315, 2133.244)	0.0177
Moderate-intensity exercise duration (min/day)	73.800 (71.623, 75.978)	88.918 (79.522, 98.313)	0.0048
HDL (mg/dL)	54.038 (53.270, 54.807)	51.457 (48.780, 54.133)	0.0462
Total cholesterol (mg/dL)	196.244 (194.468, 198.020)	198.092 (190.513, 205.671)	0.6342
Neutrophil count (× 10^3^ cells/uL)	3.925 (3.844, 4.006)	4.482 (4.259, 4.704)	< 0.0001
White blood cell count (× 10^3^ cells/uL)	6.678 (6.571, 6.785)	7.468 (7.206, 7.730)	< 0.0001
Vitamin C (mcg)	83.029 (78.710, 87.348)	69.044 (51.957, 86.130)	0.1430
Zinc (mg)	12.155 (11.716, 12.594)	10.603 (9.979, 11.227)	0.0013
Se (μg)	114.030 (111.127, 116.932)	98.522 (91.703, 105.341)	0.0003
Petunidin (mg)	0.975 (0.760, 1.190)	0.169 (0.093, 0.245)	< 0.0001
Malvidin (mg)	4.520 (3.787, 5.252)	0.759 (0.414, 1.104)	< 0.0001
Peonidin (mg)	1.478 (1.179, 1.777)	0.222 (0.135, 0.310)	< 0.0001
Luteolin (mg)	0.689 (0.617, 0.761)	0.439 (0.340, 0.537)	0.0017
Myricetin (mg)	1.565 (1.430, 1.701)	1.759 (0.998, 2.520)	0.6143
Total anthocyanidins (mg)	12.134 (10.629, 13.639)	3.432 (2.233, 4.631)	< 0.0001
PHQ-9 score	2.281 (2.181, 2.382)	15.021 (14.671, 15.371)	< 0.0001
Follow-up time from EXM (months)	124.938 (122.937, 126.939)	123.437 (120.105, 126.770)	0.2904
Education (%)			< 0.0001
Below secondary school level	5.939 (4.911, 7.165)	12.067 (8.675, 16.544)	
High school students in grades 9–11	12.145 (10.729, 13.719)	20.620 (15.593, 26.753)	
High school graduates	24.004 (21.804, 26.352)	26.241 (20.826, 32.485)	
Holders of an associate's degree	28.902 (27.076, 30.799)	29.473 (23.496, 36.251)	
Holders of a postgraduate degree	29.010 (26.346, 31.828)	11.600 (7.498, 17.521)	
Gender (%)			< 0.0001
Male	49.435 (47.853, 51.018)	35.097 (29.159, 41.535)	
Female	50.565 (48.982, 52.147)	64.903 (58.465, 70.841)	
Race (%)			0.0015
Mexican American	8.764 (6.550, 11.633)	8.527 (4.885, 14.471)	
Other Hispanic	4.954 (3.439, 7.086)	8.188 (4.620, 14.103)	
Non-Hispanic White	69.877 (64.852, 74.466)	63.008 (53.146, 71.893)	
Non-Hispanic Black	10.883 (8.617, 13.656)	17.733 (12.741, 24.140)	
Other Race—including multi-racial	5.522 (4.390, 6.924)	2.544 (0.961, 6.559)	
CKM stage (%)			< 0.0001
Stage 0	12.129 (10.852, 13.532)	0.000 (0.000, 0.000)	
Stage 1	22.095 (20.092, 24.237)	16.406 (11.110, 23.557)	
Stage 2	53.862 (51.667, 56.043)	62.152 (54.493, 69.250)	
Stage 3	3.672 (3.192, 4.221)	3.456 (2.534, 4.696)	
Stage 4	8.242 (7.306, 9.286)	17.986 (14.073, 22.700)	
Smoking (%)			< 0.0001
Yes	44.828 (42.060, 47.629)	61.827 (56.790, 66.622)	
No	55.172 (52.371, 57.940)	38.173 (33.378, 43.210)	
Alcohol use (%)			0.2636
Yes	76.033 (73.453, 78.435)	72.146 (65.007, 78.314)	
No	23.967 (21.565, 26.547)	27.854 (21.686, 34.993)	
Final mortality status (%)			0.4845
Alive	89.084 (87.762, 90.278)	87.828 (82.773, 91.551)	
Deceased	10.916 (9.722, 12.238)	12.172 (8.449, 17.227)	

Median and interquartile spacing were calculated for continuous variables, and *P* values were obtained using the Kruskal–Wallis test. For categorical variables, weighted Chi-square tests were used to obtain percentages (%) and *P* values

PIR: income-to-poverty ratio, CKM: cardiovascular–kidney–metabolic, BMI: body mass index, HDL: high-density lipoprotein

**Fig. 2:**
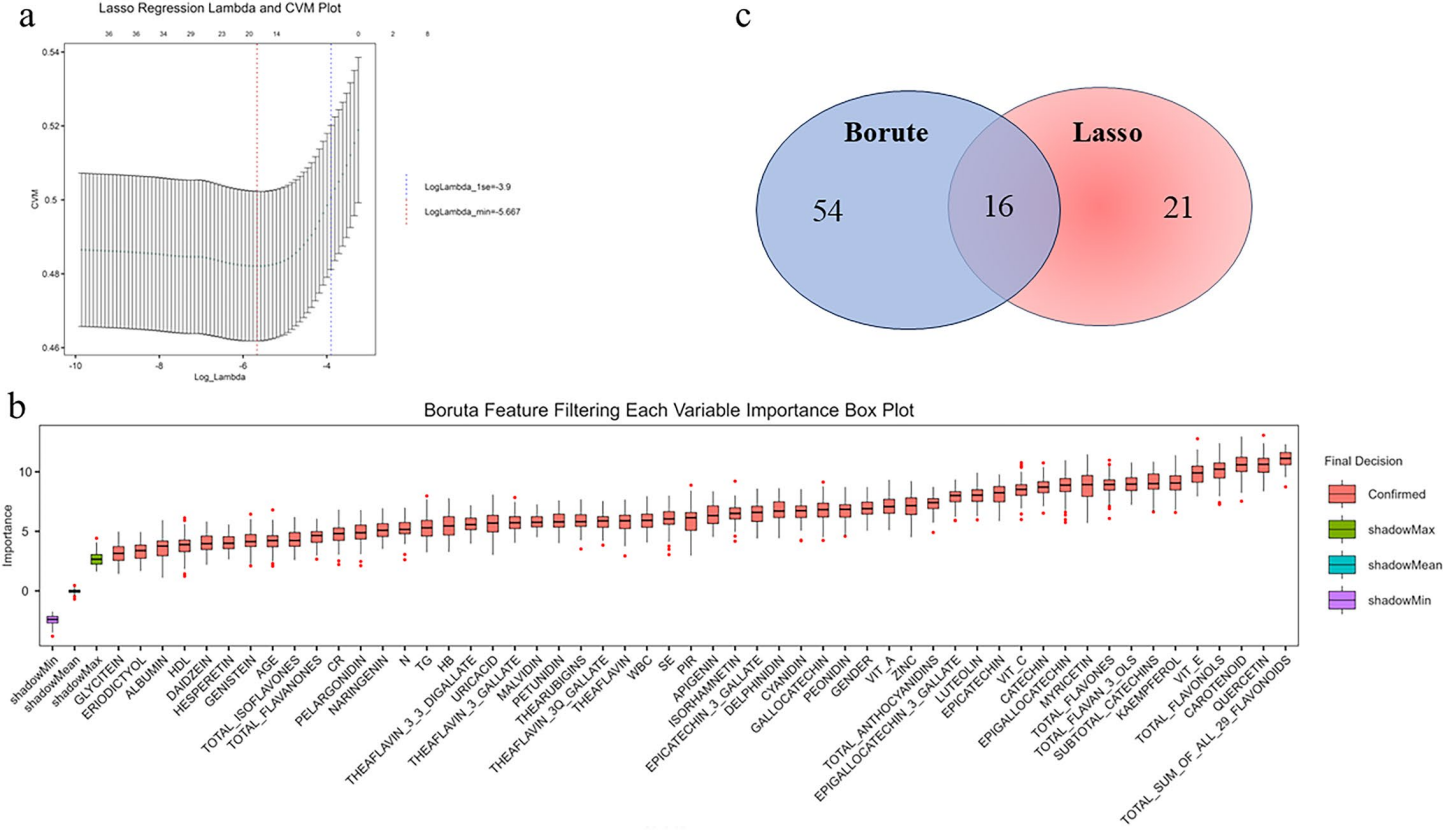
Feature selection process for variables included in the prediction model. **a** LASSO regression path showing the coefficients of variables across different values of the regularization parameter. **b** Variable importance based on the Boruta algorithm. **c** Venn diagram comparing variables selected by two different methods

Through feature selection processes, LASSO regression identified 21 candidate biomarkers linked to CKM–depression comorbidity (Fig. 2a), while the Boruta algorithm prioritized 54 variables (Fig. 2b). Intersectional analysis revealed 16 consensus predictors (Fig. 2c), comprising nutritional markers (Zinc, Se, vitamin C), anthocyanidin derivatives (total anthocyanidins, petunidin, malvidin, peonidin), flavonoid components (luteolin, myricetin), lipid profiles (HDL, TG), inflammatory indices (neutrophil, WBC), and demographic parameters (gender, PIR).

### Development and validation of the comorbidity disease prediction model

**Fig. 3:**
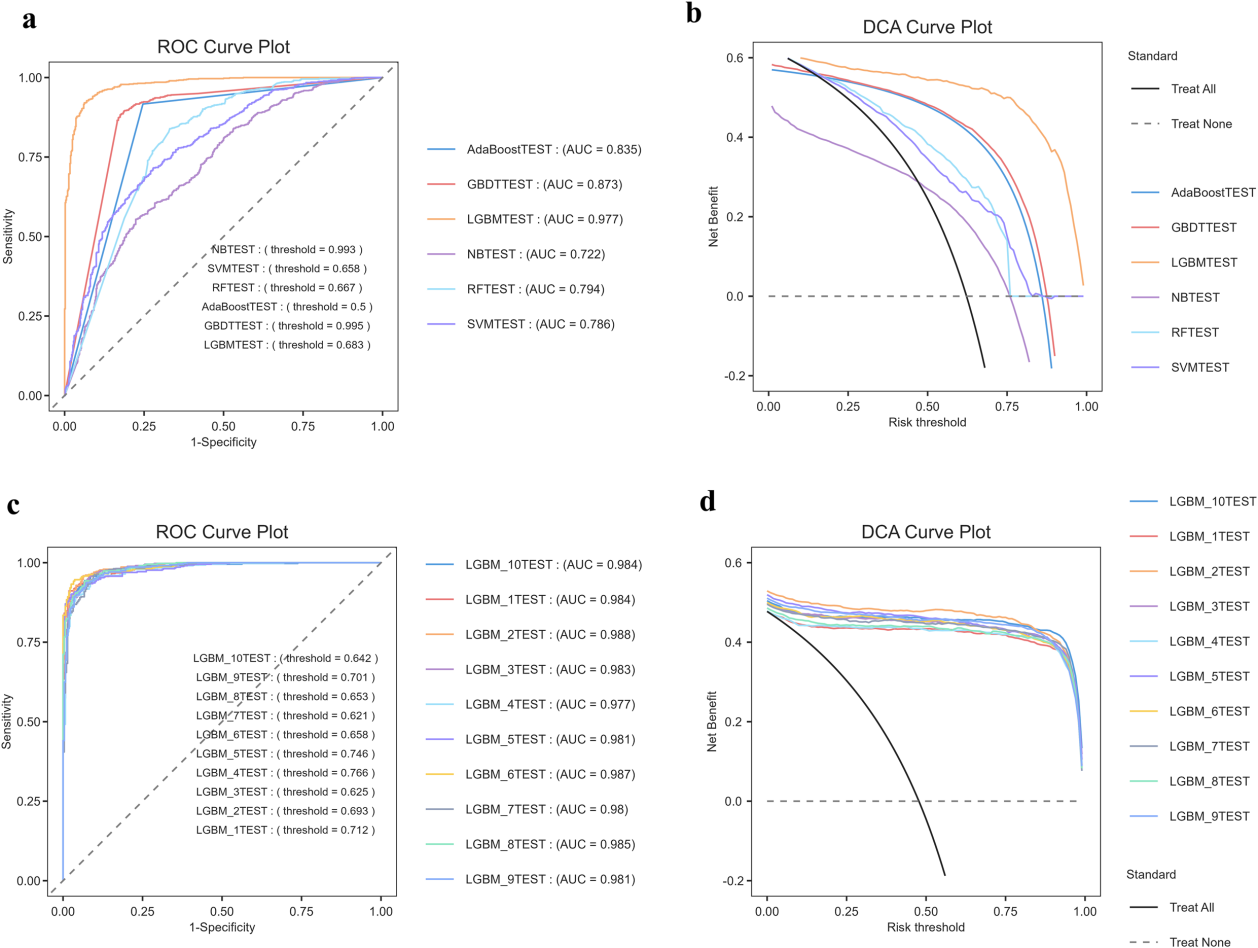
Predictive performance of the model in validation groups and cross-validation. a ROC curve for the validation set. **b** ROC curve for tenfold cross-validation. **c** Decision curve analysis (DCA) for the validation set. **d** Decision curve analysis (DCA) for tenfold cross-validation. AdaBoost: adaptive boosting, GBDT: gradient boosting decision tree, LGBM: light gradient boosting machine, NB: naive Bayes, RF: random forest, and SVM: support vector machine

The dataset was split 80/20 into training and test sets, and all six algorithms were trained on the same feature set. On the independent test set (Table 2), LGBM performed best, showing near-perfect discrimination (AUC = 0.978), the highest F1, and the most favorable error profile (high recall with acceptable specificity and the lowest FPR). GBDT and AdaBoost were slightly worse (AUCs 0.873 and 0.835). RF, SVM, and NB showed weaker generalization; notably, SVM traded specificity for recall, yielding the highest FPR. Decision-curve analysis on the test set (Fig. 3) showed the highest net benefit for LGBM across a broad threshold range (~ 0.10 to 0.70), consistently outperforming “treat-all” and “treat-none” strategies; GBDT ranked second, while RF/SVM/NB provided limited benefit only at low thresholds. The net benefit curve and ROC curve derived from tenfold cross-validation (Fig. 3) exhibit similar characteristics, confirming the robustness and clinical utility of LGBM.

**Table 2:** Indicators of six machine learning models in predicting CKM–depression comorbidity

Model name	Accuracy	Prevalence	Recall	F1-score	MCC	AUROC	Precision	Specificity	FNR	FPR
GBDT	0.860	0.622	0.920	0.891	0.697	0.873	0.863	0.759	0.080	0.241
AdaBoost	0.855	0.622	0.917	0.887	0.688	0.835	0.860	0.754	0.083	0.246
RF	0.760	0.622	0.918	0.827	0.474	0.794	0.752	0.500	0.082	0.500
LGBM	0.921	0.622	0.964	0.938	0.832	0.978	0.915	0.852	0.037	0.149
SVM	0.713	0.622	0.964	0.807	0.373	0.786	0.694	0.301	0.037	0.699
NB	0.647	0.622	0.650	0.696	0.285	0.722	0.750	0.643	0.350	0.357
Mean Scores	0.793	0.622	0.889	0.841	0.558	0.831	0.806	0.635	0.111	0.365

Accuracy, recall (sensitivity), specificity (true negative rate), precision (PPV), FNR (1—sensitivity), FPR (1–specificity), MCC, AUROC

### Importance of dietary antioxidant features interpreted by SHAP value

**Fig. 4:**
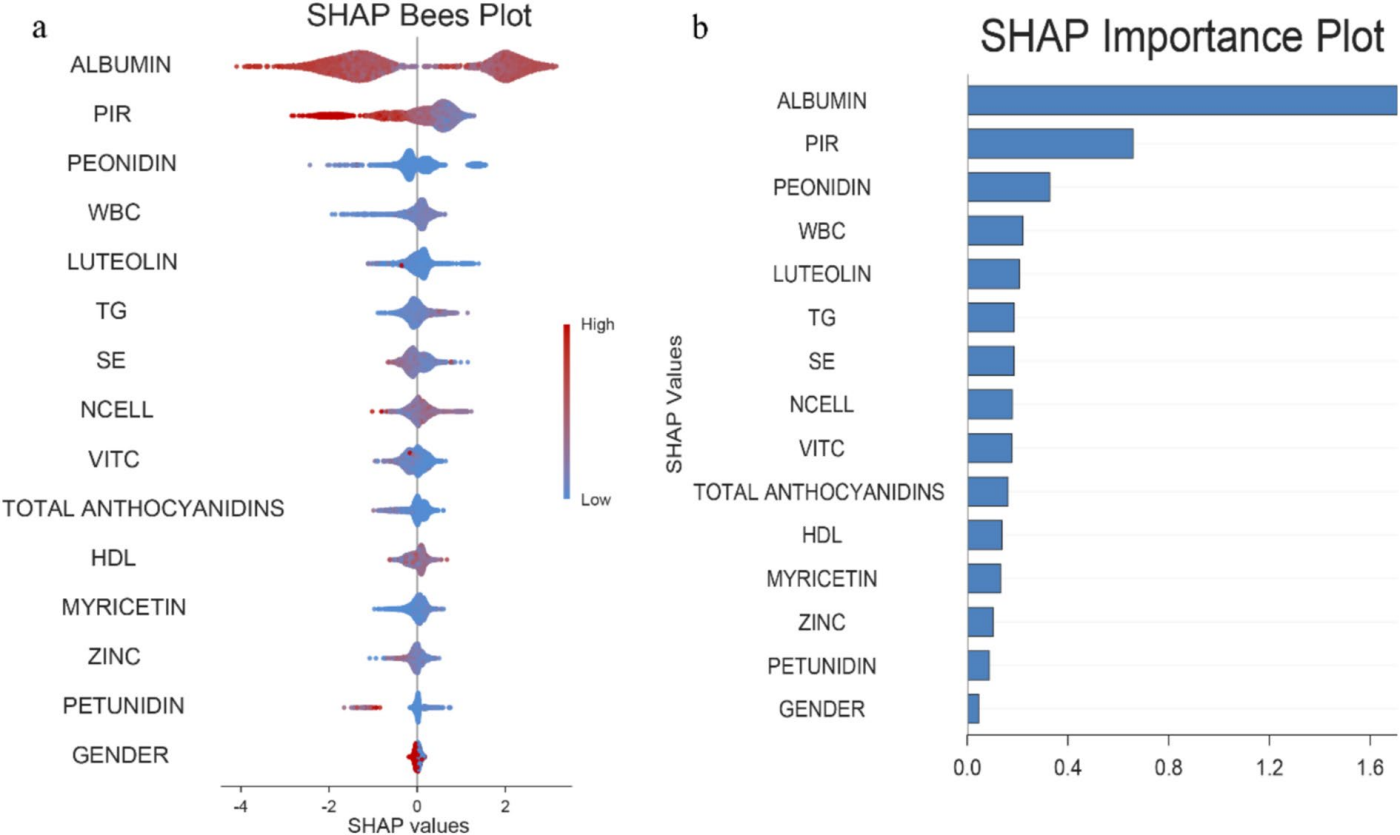
Shapley Additive exPlanations analysis of the model. **a** SHAP bees plot for associations of dietary antioxidants with CKM–depression comorbidity. **b** SHAP importance ranking plot for dietary antioxidants and CKM–depression comorbidity. PIR: income-to-poverty ratio, HDL: high-density lipoprotein, WBC: white blood cell, TG: triglyceride, NCELL: neutrophil

According to the SHAP plot (Fig. 4), the top three variables were albumin (SHAP = 1.6), PIR (SHAP ≈ 1.5), and Peonidin (SHAP ≈ 1.2), with Peonidin being the only dietary antioxidant to make it into the top three; and all the dietary antioxidants, in descending order of importance, were peonidin (SHAP ≈ 1.2), luteolin (SHAP ≈ 0.8), Se (SHAP ≈ 0.8), VITc (SHAP ≈ 0.25), total anthocyanidins (SHAP ≈ 0.25), myricetin (SHAP ≈ 0.2), zinc (SHAP ≈ 0.1) and petunidin (SHAP ≈ 0.1). These dietary antioxidants all have negative predictive effects.

### Association between dietary antioxidants and comorbidities

**Fig. 5:**
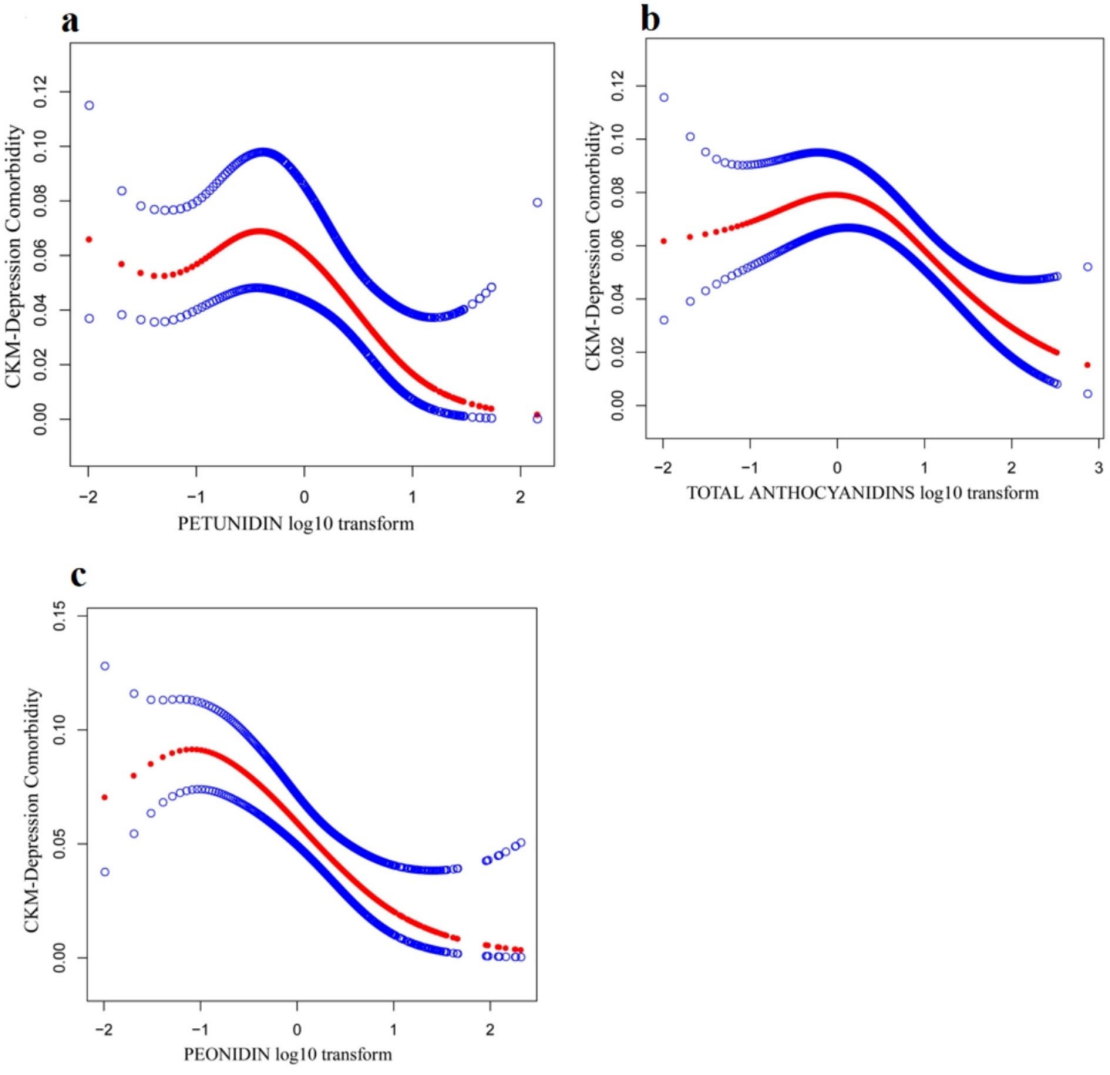
Smoothed associations (log10-transformed intake) with CKM–depression comorbidity (N = 4,996). **a** Smooth curve fitting of petunidin and CKM–depression comorbidity. **b** Smooth curve fitting of total antioxidants and CKM–depression comorbidity. **c** Smooth curve fitting of peonidin and CKM–depression comorbidity. The red line depicts the smooth curve of the log10-transformed dietary antioxidants log10 and the CKM–depression comorbidity relationship. The blue lines illustrate the 95% confidence interval. A GAMM revealed a nonlinear link between dietary antioxidants log10 and CKM–depression comorbidity

**Fig. 6:**
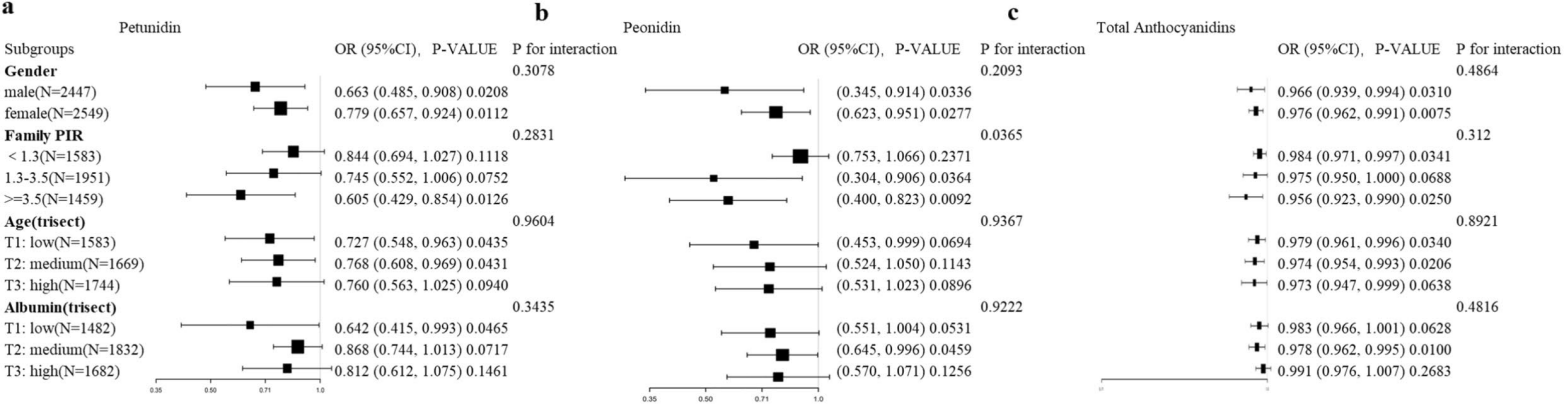
Subgroup analyses of antioxidants and CKM–depression comorbidity. **a** Subgroup analysis of petunidin comorbidity with CKM and depression. **b** Subgroup analysis of peonidin comorbidity with CKM and depression. **c** Subgroup analysis of total anthocyanidins comorbidity with CKM and depression. PIR: income-to-poverty ratio. This model adjusted for age, gender, race, family PIR, education, BMI, smoking, total energy intake, moderate-intensity exercise duration, and alcohol use

Table 3 shows a consistent inverse association between dietary antioxidants and CKM–depression comorbidity across all three models. In the fully adjusted model (Model 3), higher levels of each antioxidant were associated with lower odds of comorbidity: peonidin (OR = 0.716, 95% CI 0.572, 0.89) Petunidin (OR = 0.749, 95% CI 0.633, 0.885), and total anthocyanidins (OR = 0.973, 95% CI 0.959, 0.988). Tertile analyses yielded a similar pattern, with lower odds in the highest versus lowest tertile for each antioxidant: total anthocyanidins T3 versus T1 OR = 0.219 (95% CI 0.113, 0.424), peonidin T3 versus T1 OR = 0.421 (95% CI 0.257, 0.688), andpPetunidin T3 versus T1 OR = 0.638 (95% CI 0.425, 0.959); middle-tertile comparisons were not significant. Overall, the direction of association was consistent from Model 1 to Model 3, with expected attenuation after multivariable adjustment. In addition, a nonlinear negative association between the three and comorbidities was found using smoothed curve fitting (Fig. 5). We calculated the inflection points of the three using threshold effect analysis, *k* = −1.523 for peonidin, *k* = 0.375 for total anthocyanidins, *k* = 0.683 for petunidin, and there was a significant association between peonidin (OR = 0.558, 95% CI 0.427, 0.729) and total anthocyanidins (OR = 0.461, 95% CI 0.309, 0.690), both of which were significant on the right side of the inflection point. This suggests that peonidin and total anthocyanidins are negatively associated with comorbidities on the right side of the inflection point k. In this study, we also analyzed the multivariate mediation with total anthocyanidins, petunidin, and peonidin in comorbidities by causal mediation modeling. The results (Table 4) showed that only HDL played a mediating role in total anthocyanidins and comorbidities. After adjusting for demographic and behavioral confounders, we found that HDL showed a negative pattern of effects, with a negative total effect (OR = − 0.002302, 95% CI − 0.003339, − 0.001410) and a mediation ratio of 3.70%. In addition, as shown in Fig. 6, gender, albumin tertiles, and age had no significant effect on the negative association between dietary antioxidants and comorbidities (*P* > 0.05). As shown in Fig. 6, gender and age failed to significantly influence the negative association between the three antioxidants and comorbidities (*P* > 0.05). However, we could observe that family PIR influenced the relationship between peonidin and comorbidities (*P* = 0.0365). The odds of comorbidities were lowest in those with family PIR values between 1.3 and 3.5 (OR = 0.525, 95% CI 0.304, 0.906). For participants with a family PIR < 1.3, this association was not significant (OR = 0.896, 95% CI 0.753, 1.066).

**Table 3:** Relationship between dietary antioxidants (mg) and CKM–depression comorbidity (*N* = 4,996)

Exposure	Model 1	Model 2	Model 3
	OR (95% CI), *P* value	OR (95% CI), *P* value	OR (95% CI), *P* value
Total anthocyanidins (continuous)	0.965 (0.950, 0.981) 0.000213	0.964 (0.947, 0.981) 0.000432	0.973 (0.959, 0.988) 0.002918
Total anthocyanidins			
T1 ≤ 0.85	1.0 (ref)	1.0 (ref)	1.0 (ref)
T2 > 0.85, < 23.74	0.749 (0.612, 0.918) 0.009035	0.717 (0.586, 0.877) 0.003512	0.879 (0.714, 1.083) 0.245953
T3 ≥ 23.74	0.148 (0.077, 0.284) 0.000003	0.145 (0.075, 0.283) 0.000008	0.219 (0.113, 0.424) 0.000490
Peonidin (continuous)	0.655 (0.513, 0.837) 0.001935	0.646 (0.500, 0.834) 0.002566	0.716 (0.572, 0.897) 0.010968
Peonidin			
T1 ≤ 0.06	1.0 (ref)	1.0 (ref)	1.0 (ref)
T2 > 0.06, < 0.68	0.965 (0.692, 1.346) 0.834737	0.955 (0.678, 1.345) 0.795214	1.018 (0.705, 1.468) 0.926570
T3 ≥ 0.68	0.336 (0.209, 0.538) 0.000087	0.323 (0.198, 0.528) 0.000140	0.421 (0.257, 0.688) 0.003939
Petunidin (continuous)	0.684 (0.562, 0.833) 0.000674	0.677 (0.551, 0.832) 0.001032	0.749 (0.633, 0.885) 0.004082
Petunidin			
T1 ≤ 0.01	1.0 (ref)	1.0 (ref)	1.0 (ref)
T2 > 0.01, < 0.18	1.755 (0.838, 3.676) 0.146603	1.742 (0.819, 3.704) 0.162110	1.983 (0.890, 4.418) 0.116307
T3 ≥ 0.18	0.485 (0.328, 0.716) 0.001015	0.480 (0.319, 0.723) 0.001767	0.638 (0.425, 0.959) 0.048519

Model 1: did not adjust for covariates

Model 2: Adjusted for age, gender, and race

Model 3: adjusted for age, gender, race, family PIR, education, BMI, smoking, total energy intake, moderate-intensity exercise duration, and alcohol use

OR: odds ratio, CI: confidence intervals, PIR: income-to-poverty ratio

**Table 4:** Analysis of the mediating effect of total anthocyanidins on CKM–depression comorbidity (*N* = 4,996)

	HDL
Total effect	− 0.002 (− 0.003, − 0.001)
Mediation effect (average)	− 0.000 (− 0.000, 0.000)
Direct effect (average)	− 0.002 (− 0.003, − 0.001)
Proportion mediated (average)	0.037 (0.016, 0.094)

Model 3: adjusted for age, gender, race, family PIR, education, BMI, smoking, tTotal energy intake, moderate-intensity exercise duration, and alcohol use

HDL: high-density lipoprotein, PIR: income-to-poverty ratio

### Association of screened dietary antioxidants with all-cause mortality

**Fig. 7:**
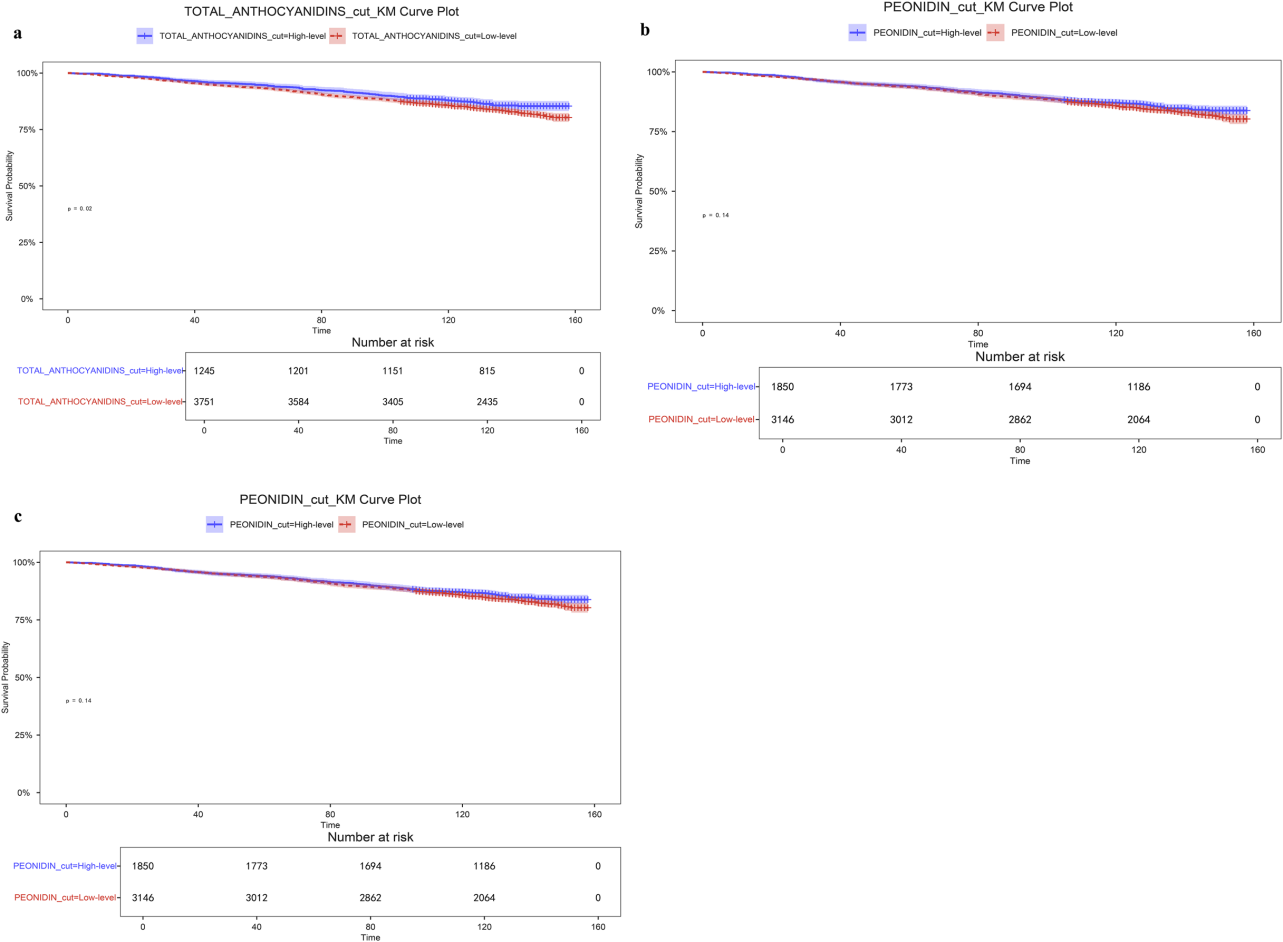
Kaplan–Meier curves for all-cause mortality by tertiles of dietary antioxidants. **a** Kaplan–Meier analysis of all-cause mortality for all participants by total anthocyanidins. **b** Kaplan–Meier analysis of all-cause mortality for all participants by petunidin trisection. **c** Kaplan–Meier analysis of all-cause mortality for all participants by peonidin

Table 5 shows the negative association between three dietary antioxidants (peonidin, petunidin, and total anthocyanidins) screened by machine learning and logistic regression, and all-cause mortality. In the multivariable Cox model, all three antioxidants showed a significant negative association with all-cause mortality. Among them, petunidin exhibited the most stable association (HR = 0.936, 95% CI 0.900, 0.975, remaining significant after permutation correction). Peonidin also showed a negative association in the multivariate model (HR = 0.964, 95% CI 0.929, 0.999). Total anthocyanidins exhibited a smaller but consistent effect size (HR = 0.993, 95% CI 0.988, 0.998), which remained significant after adjustment. In addition, the Kaplan–Meier survival curve results further assessed the relationship between these three dietary antioxidant intakes and survival outcomes. As shown in Fig. 7a and b, only participants in the total anthocyanidins and petunidin tertiary subgroups demonstrated significant survival improvement in the high-level group (P < 0.05). Notably, in Kaplan–Meier analysis, the survival difference between the high-intake and low-intake groups for peonidin did not reach statistical significance (*P* = 0.12). We modeled each antioxidant as a continuous variable using restricted cubic splines (RCS) with three knots. Likelihood ratio tests were conducted to compare the spline model against (a) the null model and (b) the linear term model, verifying (a) the overall association of the spline term and (b) the nonlinear characteristics (Supplementary Fig. 2).

**Table 5:** HRs (95% CIs) for all-cause mortality based on dietary antioxidants (*N* = 4,996)

Exposure	Model 1	Model 2	Model 3
	HR (95%CI), *P* value	HR (95%CI), *P* value	HR (95%CI), *P* value
Total anthocyanidins (continuous)	0.994 (0.990, 0.998) 0.0070	0.989 (0.983, 0.994) < 0.0001	0.993 (0.988, 0.998) 0.0082
Peonidin (continuous)	0.965 (0.930, 1.002) 0.0632	0.938 (0.894, 0.984) 0.0091	0.964 (0.929, 1.000) 0.0436
Petunidin (continuous)	0.935 (0.895, 0.977) 0.0029	0.902 (0.859, 0.947) < 0.0001	0.936 (0.900, 0.975) 0.0013

Model 1: did not adjust for covariates

Model 2: adjusted for age, gender, and race

Model 3: adjusted for age, gender, race, family PIR, education, BMI, smoking, total energy intake, moderate-intensity exercise duration, and alcohol use

HR: hazard ratio, CI: confidence intervals

### Sensitivity analysis

We built two models from screened variables. Model 1 included HDL, albumin, PIR, TG, neutrophil, WBC, and gender. Model 2 added dietary antioxidants (zinc, Se, total anthocyanidins, malvidin, luteolin, vitamin C, myricetin, petunidin, peonidin) on top of Model 1. Model 2 performed better than Model 1, indicating that dietary antioxidants add meaningful information beyond socioeconomic and health status markers (Supplementary Table 3). Moreover, the permutation-based multiple comparison correction showed that all associations remained significant except the inverse association between peonidin and all-cause mortality, which was not significant (Supplementary Table 4). *E*-value analysis was used to quantify the potential impact of residual confounding. For CKM–depression comorbidity, all three dietary antioxidants showed relatively stable associations. For all-cause mortality, *E*-values ranged from 1.09 to 1.34 (total anthocyanidins 1.09, peonidin 1.23, petunidin 1.34), suggesting that mortality outcomes were more susceptible to unmeasured confounding (Supplementary Table 5). The PH hypothesis was assessed using Schoenfeld residuals (variable-specific χ^2^ tests and diagnostic plots). As all PH tests were non-significant, no apparent time trend changes were identified (Supplementary Table 6). Besides HDL, we evaluated AIP (atherogenic index of plasma) and SII (systemic immune-inflammation index) as proxy mediators for oxidative lipid/insulin resistance and systemic inflammation, respectively. Mediation was estimated in the counterfactual framework (nonparametric bootstrap for 95% CIs). Details and results are provided in Supplementary Table 7. In addition, characteristics of participants regarding non-essential dietary antioxidants are presented in supplementary Table 8.

## Discussion

This analysis involving 4,996 subjects investigated the association between dietary antioxidant components and the CKM–depression comorbidity. Feature importance analysis based on SHAP within the optimized LGBM framework revealed that albumin was the strongest predictor, followed by family PIR, while peonidin, total anthocyanidins, and petunidin among dietary antioxidants also played crucial roles. We quantified the incremental information provided by dietary antioxidants using IDI and NRI. Consistent with our interpretation, PIR and albumin dominate, yet dietary antioxidants remain crucial, offering additional meaningful signals for assessment. Further regression results demonstrated that intake of these flavonoid subclasses negatively correlates with CKM–depression comorbidity and reduces all-cause mortality risk. It is important to note that after multiple comparison tests using the empirical *P*-value method with random permutations, most associations remained significant. Only pPeonidin failed to meet the numerous correction thresholds (without altering its negative direction of association or HR estimate), suggesting that evidence for peonidin should be considered suggestive and requires validation in independent samples. Despite adjusting for multiple socioeconomic and lifestyle covariates and incorporating physical activity and total energy intake in sensitivity analyses, residual confounding remains unavoidable. *E*-value results provide quantitative evidence: the *E*-value ≈ 2 for peonidin/petunidin in comorbid outcomes indicates that substantial unmeasured confounding would be required to nullify the association. Smaller *E*-value for mortality outcomes (≤ 1.34) suggests that weaker unmeasured confounding could explain the observed association. Therefore, we interpret the relevant findings—particularly the peonidin–mortality association—as indicative rather than causal, positioning them as suggestive evidence requiring validation in independent cohorts. Additionally, the discrepancy between the KM and Cox models stems from the fact that KM employs an unadjusted model reliant on categorical exposure variables, whereas the Cox model utilizes a continuous scale and controls for confounding factors. Our RCS and PH tests indicate that the results are not driven by model specification errors (no nonlinearity, PH holds). However, given the observational study design, these findings should still be interpreted as associative. Although HDL demonstrated a statistically significant indirect effect, its mediation proportion was small (approximately 3.7%), suggesting that this association primarily operates through pathways outside the HDL pathway. In exploratory mediation analyses using surrogate measures of insulin resistance (AIP) and systemic inflammation (SII), the ACME was not significant, with mediation proportions of 3.9% and 0.4%, respectively (see Supplementary Table 7). Collectively, these findings suggest multiple or weak mediating pathways and the limited sensitivity of surrogate markers in the NHANES study. Given the observational study design, we interpret all findings as associations rather than causal effects and call for studies directly measuring oxidative stress biomarkers (e.g., F_2_-isoprostane, 8-OHdG), insulin sensitivity indicators (e.g., fasting insulin/glucose ratio or HOMA-IR/TyG), and combined multi-mediator models.

Machine learning methods were used primarily to prioritize candidate predictors and support variable selection [28]. LGBM provided the best discrimination for CKM–depression comorbidity and consistently highlighted albumin and family PIR as dominant predictors, with anthocyanidins contributing additional but smaller predictive information (as confirmed by NRI and IDI). We therefore view ML as a complementary tool that helped identify key dietary components and contextual variables rather than as a stand-alone clinical prediction model [29].

In addition, we combined machine learning and multivariate logistic regression to determine a negative association between peonidin, petunidin, and total anthocyanidins and comorbidities. Previous studies have shown that supplementation with anthocyanidins, including peonidin and petunidin, would be beneficial for healthy adults to maintain body weight and deter obesity and its associated consequences [30]. In addition, a daily intake of 750 ml of red orange juice (containing anthocyanidins) [31] improved risk factors for metabolic syndrome, such as a significant decrease in total cholesterol (TC) and LDL levels [32]. In our study, it was observed that total anthocyanidins reduced the chances of comorbidities through HDL. This may be because the cholesterol efflux-enhancing effects of cyanidin-3-glucoside (C3G), a predominant anthocyanidin derivative, may stem from its dual regulatory actions: selective activation of liver X receptor (LXR) isoforms and transcriptional modulation of ATP-binding cassette transporters ABCA1 and ABCG1, which collectively orchestrate reverse cholesterol transport and high-density lipoprotein (HDL) biogenesis [33, 34]. HDL is known to have a reverse cholesterol-transporting effect and can attenuate atherosclerosis [35].

Furthermore, in subgroup analyses, both total anthocyanidins and petunidin maintained a stable negative association with comorbidities. However, the negative association between peonidin and comorbidities was significantly affected by family PIR (*P* for interaction < 0.05). This mechanism may hold particular importance for individuals in low-income brackets, who often have limited access to fruits and vegetables, the primary dietary sources of these beneficial compounds [36]. Our study offers insights for creating food assistance programs and public policies [37, 38]. It suggests incorporating anthocyanidin-rich foods to reduce the combined burden of CKM and depression in economically vulnerable populations.

The exact mechanism for the negative association between anthocyanidins and depression is unclear. With respect to depressive symptoms, preclinical studies indicate that anthocyanidins can reduce oxidative stress and may influence monoamine pathways, but most mechanistic data derive from animal models or in vitro assays of human enzymes [39]. Human evidence is more limited, but broadly supportive of biological plausibility. Small randomized trials of anthocyanin-rich foods or supplements have reported improvements in mood or depressive symptom scores [40], and an acute placebo-controlled study of black currant juice observed transient inhibition of platelet MAO-B activity with reductions in plasma prolactin, consistent with short-term modulation of monoamine metabolism [41, 42]. However, these interventions typically contain complex mixtures of bioactive compounds, and trials isolating individual anthocyanidin subclasses are lacking. Accordingly, we regard these mechanistic pathways as hypotheses consistent with human data rather than established causal explanations. While previous studies have focused on the relationship between total anthocyanidin intake and all-cause mortality [43], our study utilizes a comprehensive analytical approach that integrates machine learning for feature selection and multivariate logistic regression to identify specific bioactive subfractions. In vitro models have demonstrated that this mechanism may reduce monocyte adhesion to endothelial cells by reducing *E*-selectin expression. Further analysis suggests that it may also act to reduce the level of vascular endothelial growth factor, thereby effectively reducing the potential risk of plaque rupture [44].

This investigation employed a pioneering methodology combining machine learning with conventional statistical frameworks to comprehensively assess relationships between dietary antioxidant profiles and CKM–depression comorbidity, overcoming methodological constraints inherent in singular analytical approaches. Our analysis revealed novel protective associations for total anthocyanidins, petunidin, and peonidin against disease co-occurrence, establishing a scientific foundation for tailored nutritional interventions. Notably, mediation pathway analysis demonstrated that total anthocyanidins regulate HDL, thereby lowering comorbidity risk, proposing a biochemical mechanism requiring further empirical investigation.

Several methodological limitations warrant consideration. The cross-sectional design precludes causal inference. Our dietary exposures derive from 24-h recalls, which are subject to recall and social desirability bias, underreporting of energy, and substantial within-person variability; a single baseline assessment may not capture long-term patterns or changes during follow-up. Anthocyanidin intakes depend on food composition databases whose values vary by cultivar, ripeness, processing/cooking, and seasonal availability, introducing additional exposure misclassification. These errors are expected to be largely non-differential with respect to outcomes and thus likely attenuate true associations. Moreover, because missing dietary and covariate data were handled using a complete case approach, our analyses assume that, conditional on observed covariates, participants with complete data are comparable to those with missing data. If individuals with incomplete dietary data differ systematically in unmeasured factors related to antioxidant intake or CKM/mental health status, selection bias may have occurred, and our estimates may not fully generalize to all eligible NHANES participants. In primary analyses, we did not apply a log10(*x* + 1) transformation or z-score standardization to antioxidant intakes; exposures were modeled on their original mg/day scale for interpretability. Although tree-based models (e.g., LGBM) are largely insensitive to linear scaling, the lack of transformation under highly skewed distributions may reduce comparability across linear models and slightly affect probability calibration. Future work will include sensitivity analyses using log-transformed and standardized exposures. Additionally, we adopted a conservative SMOTE usage strategy (applying default parameters only on the training folds) and demonstrated consistent improvements in Recall/MCC alongside stable specificity throughout out-of-fold comparisons between unsampled and SMOTE-processed data, thereby reducing the likelihood of “artificial pattern introduction.” However, the absence of nested cross-validation for external validation constitutes a limitation of this study. Consequently, we position the machine learning results as clues for feature importance rather than clinical-grade predictions. Furthermore, given that this study's comorbidity scope encompasses patients across CKM stages 1–4, clinical manifestations may exhibit considerable heterogeneity between advanced and early stages. Nevertheless, our design emphasizes the importance of understanding comorbidities within the continuous risk spectrum framework of CKM syndrome. Previous studies indicate that individuals at stage 1 exhibit significantly elevated depression risk (HR = 1.18) compared to stage 0, with a dose–response relationship, suggesting that psychosomatic associations emerge early in the disease course [45].

## Conclusion

In this observational NHANES analysis (2007–2010), CKM–depression comorbidity was the primary outcome, and all-cause mortality was the secondary outcome. Using LASSO/Boruta screening with SHAP interpretation, followed by multivariable modeling, we found consistent inverse associations of peonidin, petunidin, and total anthocyanidins with comorbidity (all remaining significant after permutation-based multiplicity control). Regarding mortality rates, petunidin and total anthocyanidins showed a negative association, while paeoniflorin anthocyanins were no longer significant after replacement adjustment. Dose–response curves were monotonic, and proportional-hazards assumptions held. PIR and albumin were the dominant global predictors; antioxidants added modest, but stable incremental value (NRI/IDI). HDL mediation (3.7%) was statistically significant, but biologically small.

These results suggest that specific anthocyanidins may serve as dietary correlates of CKM–depression comorbidity and mortality, complementing socioeconomic and health status markers for risk stratification; however, the findings remain associational and do not establish protective or causal effects. Future work should include external validation, prospective and interventional studies, and mechanistic investigations (inflammation, redox, lipid pathways, and socioeconomic context) to clarify whether and to what extent modifying anthocyanidin intake can influence CKM–depression outcomes.

## Supplementary Information

Additional file1 (DOCX 220 KB)

## Data Availability

Researchers and data users around the world can access the survey data via the Internet (www.cdc.gov/nchs/nhanes/).

## Abbreviations

NHANES: National Health and Nutrition Examination Survey
CKM: Cardiovascular–kidney–metabolic
PIR: Income-to-poverty ratio
ML: Machine learning
HDL: High-density lipoprotein

## Declarations

### Clinical trial

Not applicable (non-trial secondary data analysis).
