# Supplementary material for: Dietary antioxidants and CKM–depression comorbidity: a primary analysis with a secondary evaluation of all-cause mortality using six machine learning algorithms
Source: Eur J Med Res. 2025 Nov 27;30:1288. doi: 10.1186/s40001-025-03524-0 (PMC12751877; doi:10.1186/s40001-025-03524-0)
Supplement: Supplementary file 1 — Additional file1 (DOCX 220 KB) [file 40001_2025_3524_MOESM1_ESM.docx]

**Missing Variable Handling**

Dietary antioxidant variables were taken from WWEIA 24-h recalls. Non-consumption on a recall day was coded as 0. Participants with missing values on any antioxidant intake variable were excluded (complete-case analysis).

**Evaluation and Explanation of Smote Sampling**

Class Imbalance Handling (SMOTE) was employed solely for oversampling in the CKM–depression comorbidity classification task. We adopted conservative SMOTE settings: k-neighbors = 5, sampling-strategy ≈ 1.0 (adjusting the minority-majority class ratio in the training set to approximately 1:1). To prevent information leakage, SMOTE was performed only within training folds. Standardization/dumb variable encoding, SMOTE, and model training were executed sequentially within each training fold, while validation/test folds remained unsampled. Due to the low positive prevalence (approximately 7.5% positive cases in unsampled data; see “Prevalence” column in Supplementary Table 1), accuracy metrics lacked discriminative power. We primarily use Recall, F1, MCC, and AUROC as key metrics, while also reporting Specificity, FNR, and FPR. All values are derived from out-of-fold predictions via stratified cross-validation. We present results for both unsampled and SMOTE-enhanced models (Supplementary Table 1 vs. Table 2) to demonstrate the gains from oversampling and its impact on specificity. This study employs conservative methods, avoiding extensive hyperparameter tuning to mitigate overfitting risks. We selected commonly used default values (k = 5, sampling ratio ≈ 1:1) and relied on consistent improvement trends across multiple algorithms for robustness validation. Future work will systematically compare different SMOTE variants (e.g., Borderline-SMOTE, SMOTE-NC) and sampling ratios within nested cross-validation frameworks.

**Hyperparameter Optimization**

The hyperparameter optimization for all machine learning models (GBDT, AdaBoost, Gaussian Naïve Bayes, Random Forest, SVM, and LGBM) was performed using Grid Search combined with 5-fold cross-validation. For each model, a parameter search grid was predefined as shown in Supplementary Table 2. The grid search exhaustively evaluated all possible parameter combinations, and the configuration achieving the highest mean AUC on the validation folds was selected as the optimal set. For models employing iterative learning (GBDT, AdaBoost, LGBM), early stopping was introduced when the validation AUC did not improve over 10 consecutive iterations. To ensure reproducibility, all random processes were controlled by setting a fixed random seed (random_state = 42). The optimized parameters summarized in Supplementary Table 2 were used for final model evaluation on the test dataset.

**Supplementary Figure 1:** Validation Set and Cross-Validation Model Calibration Curves

**
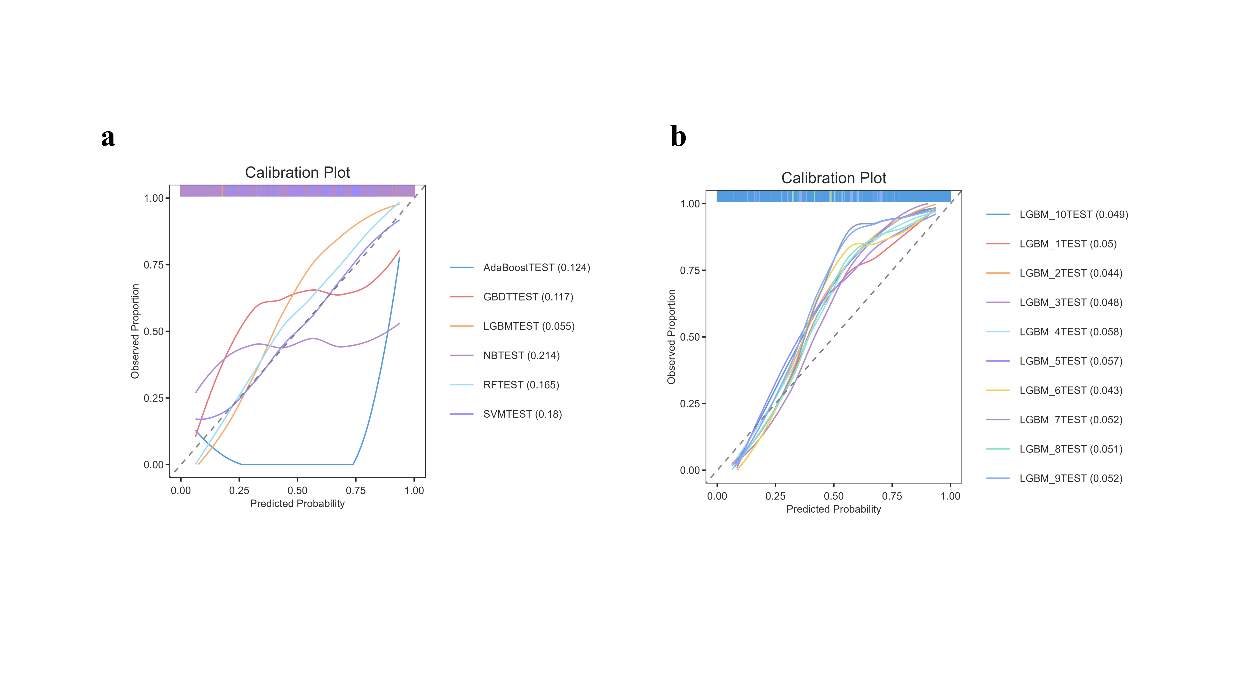
**

Supplementary Figure 1a, Calibration curve of the validation set model. Supplementary Figure 1b, Calibration curve from 10-fold cross-validation.

**Supplementary Figure 2:** Plot of unrestricted cubic splines(N = 4,996)**​**

**
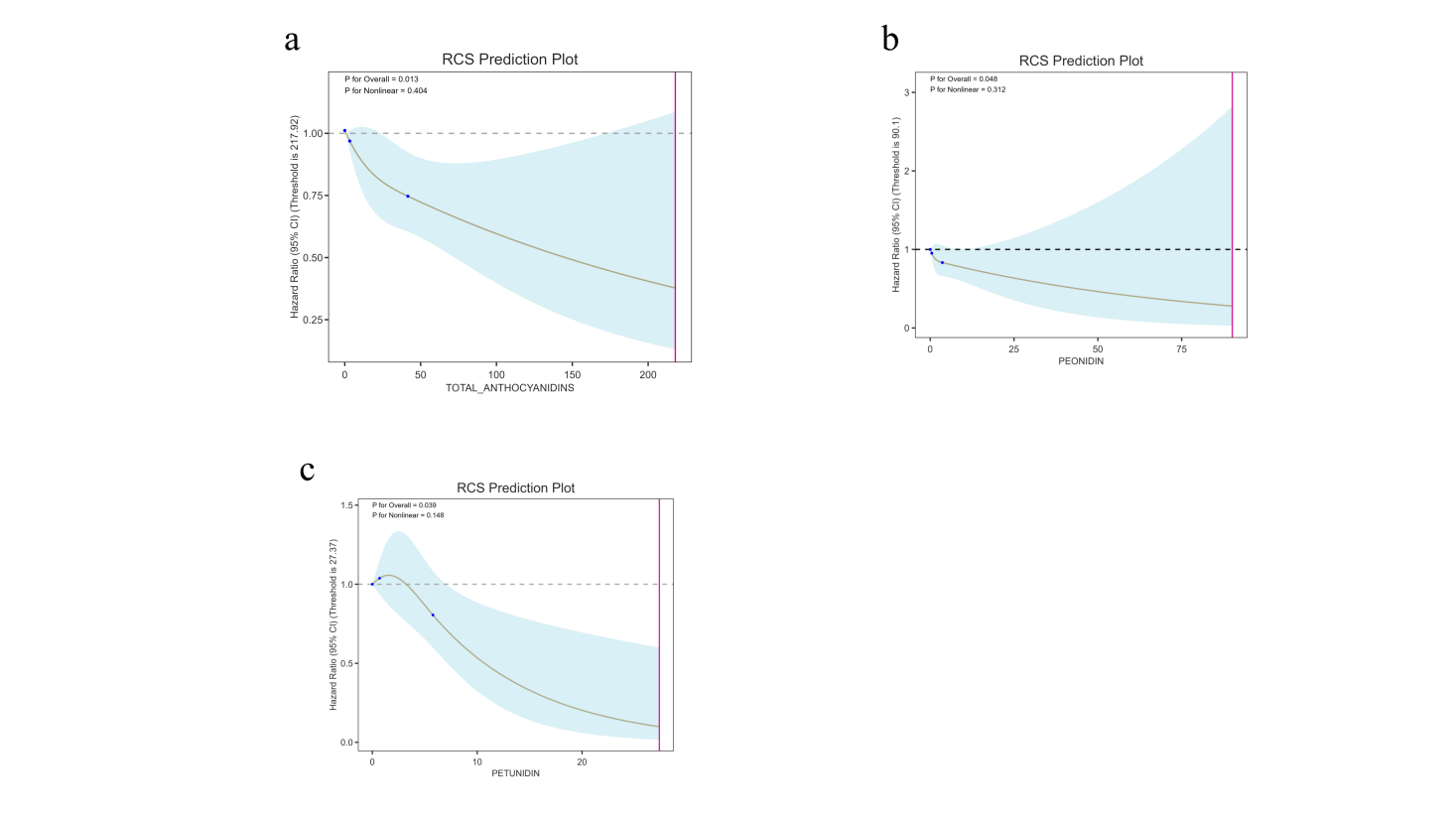
**

Supplementary Figure 2a: Unrestricted cubic spline of Total Antioxidants and all-cause mortality; Supplementary Figure 2b: Unrestricted cubic spline of Peonidin and all-cause mortality; Supplementary Figure 2c: Unrestricted cubic spline of Petunidin and all-cause mortality.

**Supplementary Table 1**: 6 unsampled machine learning models in predicting indicators of CKM-depression comorbidity.

| Model Name | Accuracy | Prevalence | Recall | F1-Score | MCC | AUC | Presicion | Specificity | FNR | FPR |
| --- | --- | --- | --- | --- | --- | --- | --- | --- | --- | --- |
| GBDT | 0.933 | 0.075 | 0.547 | 0.550 | 0.514 | 0.760 | 0.554 | 0.964 | 0.453 | 0.036 |
| AdaBoost | 0.927 | 0.075 | 0.533 | 0.523 | 0.484 | 0.746 | 0.513 | 0.959 | 0.467 | 0.041 |
| RF | 0.925 | 0.075 | 0.000 | NA | NA | 0.771 | NA | 1.000 | 1.000 | 0.000 |
| LGBM | 0.959 | 0.075 | 0.493 | 0.643 | 0.659 | 0.888 | 0.925 | 0.997 | 0.507 | 0.003 |
| SVM | 0.925 | 0.075 | 0.000 | NA | NA | 0.635 | NA | 1.000 | 1.000 | 0.000 |
| NB | 0.521 | 0.075 | 0.733 | 0.186 | 0.125 | 0.674 | 0.107 | 0.504 | 0.267 | 0.496 |
| Mean-scores | 0.865 | 0.075 | 0.384 | 0.476 | 0.445 | 0.746 | 0.525 | 0.904 | 0.616 | 0.096 |

**Supplementary Table 2:** Optimal hyperparameters of six ML models determined by Grid Search with 5-fold cross-validation.

| **Model** | Parameter optimization range | Optimal parameter |
| --- | --- | --- |
| **GBDT** | 'learning_rate':[0.001,0.005,0.01,0.05,0.1],'n_estimators':[50,100,200,500,1000],'max_depth':[2,3,4,5,6,7,8],'min_samples_split':[2,5,10,20],'min_samples_leaf':[1,2,5,10],'subsample':[0.6,0.7,0.8,0.9,1.0],'criterion':['friedman_mse','squared_error'],'random_state':[42] | learning_rate=0.01, n_estimators=500, max_depth=5, min_samples_split=5, min_samples_leaf=2, subsample=0.8, criterion='friedman_mse', random_state=42 |
| **AdaBoost** | 'n_estimators':[50,100,200,500,1000],'learning_rate':[0.001,0.005,0.01,0.05,0.1,1.0],'algorithm':['SAMME','SAMME.R'],'random_state':[42] | n_estimators=200, learning_rate=0.05, algorithm='SAMME.R', random_state=42 |
| **NB** | 'var_smoothing':[1e-12,1e-10,1e-8,1e-6,1e-4,1e-2,1e-1,1] | var_smoothing=1e-07 |
| **RF** | 'n_estimators':[100,200,500,1000],'max_depth':[3,5,7,9,None],'min_samples_split':[2,5,10],'min_samples_leaf':[1,2,4],'max_features':['sqrt','log2',None],'bootstrap':[True,False],'random_state':[42] | n_estimators=500, max_depth=7, min_samples_split=2, min_samples_leaf=1, max_features='sqrt', bootstrap=True, random_state=42 |
| **SVM** | 'C':[0.001,0.01,0.1,1,10,100],'gamma':[0.0001,0.001,0.01,0.1,1],'kernel':['linear','poly','rbf','sigmoid'],'tol':[1e-4,1e-3,1e-2],'random_state':[42] | C=1, gamma=0.01, kernel='rbf', tol=0.001, random_state=42 |
| **LGBM** | 'boosting_type':['gbdt','dart'],'learning_rate':[0.001,0.005,0.01,0.05,0.1],'n_estimators':[50,100,500,1000,1500],'num_leaves':[15,30,45,60],'max_depth':[1,3,5,7,9],'min_child_samples':[5,10,20,30],'subsample':[0.6,0.7,0.8,0.9,1.0],'colsample_bytree':[0.6,0.7,0.8,0.9,1.0],'reg_alpha':[0,0.1,0.5,1,2],'reg_lambda':[0,0.1,0.5,1,2],'random_state':[42] | boosting_type='gbdt', learning_rate=0.001, n_estimators=10, num_leaves=30, max_depth=1, min_child_samples=20, subsample=0.8, colsample_bytree=0.8, reg_alpha=0.1, reg_lambda=1.0, objective='binary', early_stopping_rounds=10, random_state=42 |

All six machine learning models were optimized using Grid Search with 5-fold cross-validation to select the best-performing hyperparameters. For models supporting iterative training (GBDT, AdaBoost, LGBM), early stopping was applied when the validation AUC failed to improve for 10 consecutive rounds. A fixed random seed (random_state = 42) was used for all models to ensure reproducibility.

**Supplementary Table 3**: Incremental Value of Antioxidants: NRI and IDI

| Row Name | Value | SE | Z-Value | CI (95Lower) | CI (95Upper) | ***P***-value |
| --- | --- | --- | --- | --- | --- | --- |
| NRI | 0.254 | 0.047 | 5.347 | 0.161 | 0.347 | <0.001 |
| IDI | 0.008 | 0.001 | 6.223 | 0.006 | 0.011 | <0.001 |

Model 1 includes HDL, Albumin, PIR, TG, Ncell, WBC, and Gender

Model 2 includes HDL, Albumin, PIR, TG, Ncell, WBC, and Gender, Zinc, Se, Total Anthocyanidins, Malvidin, Luteolin, VitC, Myricetin, Petunidin, and Peonidin

Abbreviations: PIR, Income-to-Poverty Ratio

**Supplementary Table 4:** Calculate multiple comparison empirical ***P***-values for primary outcomes using the random exchange method.

| **Outcome** | **Exposure** | **P (observe)** | **P (permute)** | **Exp (Coeff)** | **95% Low** | **95% Upp** |
| --- | --- | --- | --- | --- | --- | --- |
| Comorbidity of CKM and Depression | Total anthocyanidins | 0.000009 | 0.001998 | 0.977 | 0.967 | 0.988 |
| Comorbidity of CKM and Depression | Petunidin | <0.000001 | 0.000999 | 0.765 | 0.662 | 0.883 |
| Comorbidity of CKM and Depression | Peonidin | 0.000002 | 0.001998 | 0.717 | 0.609 | 0.844 |
| All causes of death | Total anthocyanidins | 0.010180 | 0.046953 | 0.995 | 0.992 | 0.998 |
| All causes of death | Petunidin | 0.010103 | 0.047952 | 0.947 | 0.909 | 0.987 |
| All causes of death | Peonidin | 0.079383 | 0.303696 | 0.979 | 0.956 | 1.002 |

This model adjusted for age, gender, race, education, family PIR, BMI, Total energy intake, Moderate-intensity exercise duration, smoking, and alcohol use.

Abbreviations: PIR, Income-to-Poverty Ratio

**Supplementary Table 5:** Results of E-value analysis

| **Exposure** | **Outcome** | **Estimate(95%CI)** | **E-value** | **RR.e-u** | **RR.u-d** |
| --- | --- | --- | --- | --- | --- |
| Total Anthocyanidins | CKM-depression comorbidity | 0.974 (0.974,0.988) | 1.20 | 1.17 | 1.23 |
| Peonidin | CKM-depression comorbidity | 0.720 (0.577, 0.899) | 2.14 | 1.68 | 3.35 |
| Petunidin | CKM-depression comorbidity | 0.750 (0.635, 0.886) | 2.00 | 1.60 | 3.02 |
| Total Anthocyanidins | all-cause mortality | 0.993 (0.988, 0.998) | 1.09 | 1.19 | 1.05 |
| Peonidin | all-cause mortality | 0.964 (0.929, 1.000) | 1.23 | 1.16 | 1.35 |
| Petunidin | all-cause mortality | 0.936 (0.900, 0.975) | 1.34 | 1.12 | 2.48 |

E-value represents the minimum strength of unmeasured confounding needed to explain away the observed association. RR.e–u: association between exposure and unmeasured confounder; RR.u–d: association between unmeasured confounder and outcome.

**Supplementary Table 6:** Results of the proportional hazards assumption

| **Variable Name** | **chisq** | **df** | ***P*-value** |
| --- | --- | --- | --- |
| Total Anthocyanidins | 0.249 | 1 | 0.618 |
| Peonidin | 1.961 | 1 | 0.161 |
| Petunidin | 0.753 | 1 | 0.385 |

**Supplementary Table 7:** Analysis of the mediating effect of Total Anthocyanidins on CKM–depression comorbidity(N = 4,996).

|  | AIP | SII |
| --- | --- | --- |
| **Total effect** | −0.000360 (−0.000534, −0.000198) | −0.000360 (−0.000534, −0.000198) |
| **Mediation effect (average)** | −0.000014 (−0.000036, 0.000002) | −0.000001 (−0.000016, 0.000009) |
| **Direct effect (average)** | −0.000346 (−0.000517, −0.000189) | −0.000359 (−0.000531, −0.000198) |
| **Proportion mediated (average)** | 0.0387 (−0.0065, 0.1144) | 0.0040 (−0.0260, 0.0477) |

Abbreviation: AIP, Lipid Atherosclerosis Index; SII, Systemic Inflammation Index; PIR, Income-to-Poverty Ratio

Model 3: adjusted for age, gender, race, family PIR, education, BMI, smoking, Total energy intake, Moderate-intensity exercise duration, and alcohol use

**Supplementary Table 8:** Weighted baseline characteristics of non-key dietary antioxidants among participants after CKM depression comorbidity classification(N = 4,996).

| **CKM-depression comorbidity** | | | |
| --- | --- | --- | --- |
| Characteristics | No | Yes | ***P***-value |
| **Vit A (RAE)** | 626.436 (601.481,651.391) | 546.466 (457.166,635.766) | 0.1059 |
| **Vit E (mcg)** | 7.908 (7.650 ,8.166) | 6.468 (5.915 ,7.022) | 0.0002 |
| **Carotenoid (mg)** | 9621.757 (8975.849,10267.664) | 7425.111 (5790.963,9059.260) | 0.0201 |
| **Daidzein (mg)** | 0.654 (0.504,0.803) | 0.342 (0.106,0.577) | 0.0239 |
| **Genistein (mg)** | 0.935 (0.671,1.200) | 0.419 (0.143,0.695) | 0.0081 |
| **Glycitein (mg)** | 0.132 (0.090 ,0.174) | 0.035 (0.011 ,0.060) | 0.0002 |
| **Cyanidin (mg)** | 2.279 (1.922,2.636) | 1.259 (0.731,1.788) | 0.0022 |
| **Delphinidin (mg)** | 1.356 (1.057,1.656) | 0.344 (0.166,0.523) | <0.0001 |
| **Pelargonidin (mg)** | 1.526 (1.189 ,1.864) | 0.678 (0.157 ,1.199) | 0.0185 |
| **Catechin (mg)** | 8.132 (7.585,8.680) | 5.942 (4.697,7.187) | 0.0056 |
| **Epigallocatechin (mg)** | 17.918 (15.549,20.288) | 18.515 (10.472,26.558) | 0.8832 |
| **Epicatechin (mg)** | 10.485 (9.850,11.120) | 9.512 (7.180,11.845) | 0.4427 |
| **Epicatechin-3-gallate (mg)** | 11.815 (10.213,13.416) | 11.935 (6.825,17.044) | 0.9631 |
| **Epigallocatechin-3-gallate (mg)** | 31.078 (26.964,35.193) | 34.010 (18.113,49.907) | 0.7131 |
| **Theaflavin (mg)** | 1.806 (1.545,2.066) | 1.510 (0.968,2.052) | 0.3113 |
| **Thearubigins (mg)** | 103.309 (89.260,117.357) | 106.270 (60.024,152.516) | 0.8989 |
| **Eriodictyol (mg)** | 0.211 (0.184,0.238) | 0.163 (0.070,0.257) | 0.3172 |
| **Hesperetin (mg)** | 8.927 (7.943,9.910) | 8.837 (4.313,13.362) | 0.9699 |
| **Naringenin (mg)** | 3.099 (2.711,3.486) | 3.525 (0.978,6.073) | 0.7457 |
| **Apigenin (mg)** | 0.207 (0.184,0.230) | 0.176 (0.130,0.222) | 0.2765 |
| **Isorhamnetin (mg)** | 0.946 (0.871 ,1.021) | 0.742 (0.586 ,0.899) | 0.0294 |
| **Kaempferol (mg)** | 5.058 (4.722 ,5.394) | 4.662 (3.338 ,5.987) | 0.5513 |
| **Quercetin (mg)** | 12.264 (11.546,12.983) | 11.528 (9.056,14.000) | 0.5680 |
| **Theaflavin-3,3’-digallate (mg)** | 1.996 (1.708,2.284) | 1.666 (1.069,2.263) | 0.3052 |
| **Theaflavin-3q’-gallate (mg)** | 1.686 (1.441,1.931) | 1.412 (0.900,1.923) | 0.3201 |
| **Theaflavin-3’-gallate (mg)** | 1.443 (1.236,1.650) | 1.202 (0.775,1.630) | 0.2970 |
| **Gallocatechin (mg)** | 1.938 (1.685,2.190) | 1.763 (1.099,2.426) | 0.6085 |
| **Subtotal_Catechins (mg)** | 81.366 (72.100,90.633) | 81.676 (48.684,114.669) | 0.9852 |
